# A Retrospective Review of Tumor Lysis Syndrome Associated With Colorectal Cancer

**DOI:** 10.7759/cureus.8257

**Published:** 2020-05-24

**Authors:** Zaid Ansari, Dawood Findakly, Jue Wang

**Affiliations:** 1 Internal Medicine, Creighton University School of Medicine/St. Joseph's Hospital and Medical Center, Phoenix, USA; 2 Internal Medicine, Creighton University Arizona Health Education Alliance/Valleywise Health Medical Center, Phoenix, USA; 3 Genitourinary Oncology, Creighton University School of Medicine/University of Arizona Cancer Center at Dignity Health, Phoenix, USA

**Keywords:** tumor lysis in solid tumors, colorectal cancer, spontaneous tls (stls), tumor lysis syndrome

## Abstract

Tumor lysis syndrome (TLS) is a life-threatening oncologic condition that is most commonly linked with hematologic malignancies and uncommonly seen in solid tumors, including colorectal cancer (CRC). Therefore, a lack of awareness regarding TLS in CRC could lead to significant morbidity and mortality. This study aims to explore the clinical characteristics and outcomes of TLS in patients with CRC.

A systematic review of the literature was performed by searching PubMed using the keywords “tumor lysis syndrome” and “colorectal cancer”. The English-language case reports and abstracts that the search results yielded were reviewed, and additional articles of interest were identified from reference lists. Information regarding the patients (age at diagnosis, presentation, and comorbidities), the tumors (histology, grade, and stage), radiologic investigations, treatment modalities (surgery, radiation, and systemic therapy), and the outcomes (response, adverse events) were recorded, when available. Descriptive statistics, such as frequency counts, medians, and ranges, were used to characterize the pooled sample.

Nine case reports of TLS in CRC were identified in the literature; one additional case was added from our patient database. The median age of these patients was 58.5 years (range: 42-82 years) with 70% of these patients being male. Of note, 100% of these patients had metastatic colon cancer and 80% had metastatic involvement of the liver; 70% of these cases were associated with therapy-induced TLS with the median time-to-event being three days (range: 18 hours-30 days) after receiving chemotherapy. When looking at laboratory parameters, uric acid and lactate dehydrogenase (LDH) were consistently elevated in all the cases, but 50% of the cases had hyperkalemia and 50% had hyperphosphatemia. Treatment of TLS included supportive measures with IV hydration. Five out of 10 patients received urate oxidase and only one underwent hemodialysis. The overall mortality was 60%.

TLS can occur with CRCs that demonstrate a high tumor burden. While most cases are associated with therapy, some cases are spontaneous in nature. Keeping in mind the high mortality associated with TLS, physicians should have a high degree of suspicion and should be aware of the fatal complications associated with TLS. Timely implementation of prophylactic and therapeutic measures including IV hydration as well as the use of xanthine oxidase inhibitors such as allopurinol can be life-saving in these cases.

## Introduction and background

Tumor lysis syndrome (TLS) is an acute life-threatening condition in oncology patients. It occurs in rapidly proliferating tumor cells through rapid tumor cell lysis either spontaneously or following anti-cancer therapy. TLS is characterized by a constellation of metabolic and electrolyte abnormalities, including hyperkalemia, hyperphosphatemia, hyperuricemia, and hypocalcemia. These could consequently evolve into widespread severe end-organ damage, leading to acute kidney injury (AKI), cardiac arrhythmia, seizures, and eventually death. While it is widely known to be highly prevalent in hematologic malignancies, it is thought to infrequently occur in solid tumors, particularly colorectal cancer (CRC). The rarity of TLS has made it challenging to determine its true incidence in CRC patients. In this article, we aim to explore the clinical characteristics and outcomes of TLS in patients with CRC [1].

## Review

Materials and methods

Study Design and Literature Search Strategy

This was a systematic review of published reports in patients with CRC who were diagnosed with TLS, with the addition of one more patient from our hospital records. PubMed was searched with the keywords “tumor lysis syndrome” and “colorectal cancer”. The resulting English-language case reports and abstracts were reviewed and additional articles of interest were identified from reference lists.

Data Collection and Statistical Analysis

Information regarding the patients (age at diagnosis, presentation, and comorbidities), the tumors (histology, grade, and stage), radiologic investigations, treatment modalities (surgery, radiation, and systemic therapy), and the outcomes (response, adverse events) were recorded, when available. Descriptive statistics such as frequency counts, medians, and ranges were used to characterize the pooled sample.

Results

Nine case reports of TLS in CRC were identified in the literature; one additional case was added from our patient database [2-9]. Patient demographics and disease characteristics such as metastasis, time of onset of TLS from cancer treatment, management, and outcomes of TLS are summarized in the table below (Table 1). The median age of these patients was 58.5 years (range: 42-82 years) with 70% of these patients being male. All the patients in this case series had metastatic colon cancer with 80% having metastatic involvement of the liver. And 80% of these cases were associated with therapy-induced TLS with the median time-to-event being three days (range: 18 hours-30 days) after receiving chemotherapy. Treatments preceding TLS varied and included irinotecan, 5-FU, bevacizumab, capecitabine, panitumumab, regorafenib, cetuximab, and oxaliplatin. When looking at laboratory parameters, uric acid and LDH were consistently elevated in all the cases, but only 50% of cases had hyperkalemia and 50% of cases had hyperphosphatemia. Treatment of TLS included supportive measures with IV hydration. Five out of 10 patients received urate oxidase and only one underwent hemodialysis. Overall mortality was 60%.

**Table 1:** List of cases reviewed TLS: tumor lysis syndrome; UA: uric acid; LDH: lactate dehydrogenase; ULN: upper limit of normal; Y: yes; N: no; NR: not reported

Author (year)	Patient age	Sex	Spontaneous vs chemotherapy	Outcome	Time from treatment to TLS	UA >7.2	LDH >2X ULN	Hypocalcemia	Hyperkalemia	Hyperphosphatemia
Kim et al. (2014) [2]	59	M	Folfox	Survival	3 days	Y	Y	Y	Y	N
Vaisban et al. (2003) [3]	82	F	Spontaneous	Survival	N/A	Y	Y	Y	NR	N
Boisseau et al. (1996) [4]	42	F	Irinotecan	Death	1 week and 1 day	Y	Y	Y	Y	Y
Krishnan et al. (2008) [5]	64	M	Cetuximab	Death	18 hours	Y	Y	Y	Y	Y
Oztop et al. (2004) [6]	66	M	Irinotecan, 5-FU	Death	3 days	Y	Y	Y	N	Y
Shah et al. (2014) [7]	58	M	Spontaneous	Survival	N/A	Y	Y	N	N	N
Farooqi et al. (2015) [8]	52	M	Regorafenib	Death	1 week	NR	NR	Y	Y	Y
Hentrich et al. (2008) [9]	72	M	Irinotecan	Death	2 days	Y	Y	NR	NR	Y
Added new case	51	M	Capecitabine	Survival	4 weeks and 2 days	Y	Y	N	N	N

Discussion

TLS is an oncological emergency that results from massive cytolysis of malignant cells with a sudden release of their cellular contents, such as intracellular ions and metabolic by-products, into the systemic circulation. This syndrome is common in tumors with rapid cell turnover and growth rates, and in bulky tumors with high sensitivity to antineoplastic treatments. It is, therefore, a well-recognized clinical problem in hematological malignancies. It is rarely observed in solid tumors [10]. When seen in solid tumors, it is usually in cases of high tumor burden. In the recent past, with the advent of novel chemo/radiotherapy options, we have seen TLS associated with solid tumors that are highly sensitive to these therapies.

There have been some reviews of TLS associated with different solid tumors such as urothelial cancer, germ cell tumors, etc [11,12]. We have compiled a collection of cases of TLS associated with CRC. In keeping with the trend seen with other solid tumors, all of the cases that we reviewed had a high tumor burden. But only two of the 10 cases had spontaneous tumor lysis. This explains why TLS was not previously associated with colorectal malignancies; however, with newer and more aggressive chemotherapeutic options, we have seen an increase in the number of cases.

Secondly, a high mortality of 60% was observed in CRCs with TLS, as compared to the 21% overall in-hospital mortality of TLS [13]. This is an important reason why clinicians should be made aware of the potentially fatal outcomes of patients with metastatic colon cancer. Multiple factors could be responsible for the higher mortality seen. Patients with higher tumor burden may have poorer overall health due to cachexia, multiple organ system involvements, or they may simply have more aggressive forms of cancer. A lack of awareness among clinicians about the possibility of TLS in CRC could also potentially preclude these patients from getting timely therapies like urate oxidase, aggressive IV hydration, and allopurinol, which may alter the course of the disease and eventual outcome. Although five patients received urate oxidase in our review, we do not have the data related to the timing of administering of urate oxidase; it is unclear if earlier vs late administration can affect outcomes.

As expected, LDH and uric acid were elevated in all cases. Of note, while phosphate was elevated only in 50% of the case, none of these patients survived. Hyperphosphatemia is known to express in the tubules and cause AKI, and with the widespread use of hypouricemic agents, calcium phosphate deposition (nephrocalcinosis) instead of hyperuricemia is the more common cause of AKI [14,15].

Our review highlights a rare oncologic emergency associated with CRC. Although there are not many cases reported, we suspect the incidence is higher than what is apparent at present, and raising awareness among physicians can help diagnose and treat a life-threatening complication. Further investigation is required to better understand the incidence and risk factors of TLS in CRC to provide recommendations for prophylaxis.

## Conclusions

TLS can occur in CRCs with a high tumor burden. While most cases are associated with therapy, some cases are spontaneous in nature. Given the high rate of mortality associated with TLS, physicians should maintain a high index of suspicion and should be aware of the fatal complications associated with TLS. The implementation of prophylactic and therapeutic measures including IV hydration as well as the use of xanthine oxidase inhibitors such as allopurinol in a prompt and timely manner can prove to be life-saving measures in these cases.
